# Effect of Whitening Toothpaste on Surface Roughness and Colour Alteration of Artificially Extrinsic Stained Human Enamel: In Vitro Study

**DOI:** 10.3390/dj10100191

**Published:** 2022-10-13

**Authors:** Sarat Suriyasangpetch, Pimduean Sivavong, Boondarick Niyatiwatchanchai, Thanaphum Osathanon, Puliwan Gorwong, Chawalid Pianmee, Dusit Nantanapiboon

**Affiliations:** 1 Department of Operative Dentistry, Faculty of Dentistry, Chulalongkorn University, Bangkok 10330, Thailand; 2 Dental Stem Cell Biology Research Unit and Department of Anatomy, Faculty of Dentistry, Chulalongkorn University, Bangkok 10330, Thailand; 3 Dental Material Research and Development Center, Faculty of Dentistry, Chulalongkorn University, Bangkok 10330, Thailand; 4 Dental Department, Surin Hospital, Surin 32000, Thailand

**Keywords:** whitening toothpaste, surface roughness, colour, time, in vitro

## Abstract

Objective: The aim of this study was to compare five toothpastes in terms of alteration of surface roughness and colour of red-wine-stained human enamel over time after brushing simulation. Methods: Stained specimens were randomly divided into five groups (*n* = 8): Oral-B Gum and Enamel (C), ZACT Stain Fighter (ZW), Colgate Optic White Volcanic Mineral (CW), Oral-B 3D White Luxe Fresh Breath (3DW), and Thepthai (TW). The colour and surface roughness of the specimens were measured after brushing simulation for four durations; two weeks, one month, six months, and twelve months. Abrasive particles in toothpaste were inspected under a scanning electron microscope. Results: Surface roughness was increased in the specimens that underwent brushing simulation in all groups (*p* < 0.05). ZW (6.33 ± 0.98 μm) exerted the most changes in surface roughness for all durations. Other groups showed similar surface roughness at each time point. ΔE_00_ and ΔL were increased in all groups until twelve months; however, there were no significant differences between C (ΔE_00_ = 30.17 ± 4.16, ΔL = 38.19 ± 4.34), CW (ΔE_00_ = 24.25 ± 10.52, ΔL = 31.12 ± 11.00), and TW (ΔE_00_ = 29.00 ± 3.96, ΔL = 36.68 ± 4.53) at any time period. Moreover, ZW (ΔE_00_ = 45.6 ± 8.01, ΔL = 53.03 ± 6.71) and 3DW (ΔE_00_ = 43.6 ± 7.33, ΔL = 51.03 ± 6.47) exhibited a substantial change and were statistically differed from the other groups after four-weeks. Various characteristics of abrasive particles were inspected under SEM. Conclusion: All five toothpastes increased the surface roughness altered the colour of red wine-stained human enamel over time.

## 1. Introduction

The term “extrinsic tooth discolouration” refers to a stain on the outer tooth surface of teeth resulting from food and beverages, such as coffee and wine, or cigarettes. These variables enable the adsorption of pigments, including tannins and polyphenols, on the tooth surface. In the extrinsic stain adhesion mechanism, calcium ions from the enamel crystallites are dissolved in saliva, leaving phosphate ions on the surface of the tooth. The enamel surface is negatively charged by the remaining phosphate ions. The positive charge of calcium counter ions forms a layer referred to as the “stern layer and hydration layer” as soon as saliva comes into contact with a negatively charged surface. Then, the protein in saliva forms acquired pellicles on the tooth surface. Negative ions from acidic protein groups, such as carboxyl, phosphate, or sulphate, are adsorbed by positive calcium counter ions, forming an electrostatic “calcium bridge”, whereas base proteins have positive ions that push the same calcium ions in the stern layer and bind directly with the phosphate group on the enamel surface. Then, chromogens adsorb directly into the acquired pellicles [1]. Moreover, the degree of discolouration is affected by the patient’s oral hygiene, smoking habits, consumption behaviour, and chromogenic bacteria [2]. Additionally, the acidity of beverages increases the risk of staining due to a demineralization on the outer surface of the tooth structure [3]. The eroded tooth surface enables food pigments to penetrate, resulting in interior discoloration [2]. Consequently, stains become ingrained and difficult to remove.

Tooth brushing is one of the most convenient procedures to remove plaque and extrinsic stains on a regular basis. Whitening toothpaste is an optional method that has become increasingly popular in the market. Whitening toothpaste is able to brighten the tooth structure through mechanical and chemical reactions. Stains can be removed through a mechanical process achieved with the use of abrasive agents, such as charcoal, hydrated silica, or alumina particles. Moreover, the particle size, morphology, hardness, and quantity of abrasive agents, as well as the amount of toothpaste, have a significant influence on the effectiveness of stain removal [4,5,6]. Furthermore, chemical agents, such as enzymes, antitartar, and peroxide, can help with stain removal. However, the use of peroxide chemical components remains highly controversial in terms of safety and side effects [7]. Therefore, antitartar agents were introduced, such as sodium hexametaphosphate and disodium pyrophosphate, which are commonly added to toothpaste, as they can bind with protein pellicles, preventing incoming stain-attached salivary proteins from adhering to the enamel [4].

Studies have indicated that the abrasive particles and chemical agents may cause damage to the tooth structure [8,9]. The duration of use of whitening toothpaste may affect tooth surface roughness, so the recommended period is still debatable, as some studies have indicated an increase in surface roughness, whereas others have reported a decrease in surface roughness in various periods of time [10,11,12,13,14]. However, only a few studies have simultaneously evaluated both the whitening effect and the tooth surface roughness over a long period of time [14,15,16]. Therefore, in the present study, we aimed to determine the effects of whitening toothpaste on the surface roughness and colour alteration of red wine-stained human enamel over time.

## 2. Materials and Methods

### 2.1. Sample Size Calculation

The sample size was calculated using G*Power software (version 3.1, Heinrich-Heine Dusseldorf University, Dusseldorf, Germany). The parameters were set with a 95% confidence interval, an 80% power, and 0.54 effect size values. The reference values were taken from a previous study by Koc Vural U et al. [14]. the sample size was calculated as eight specimens per group.

### 2.2. Tooth Collection

A total of 40 human permanent maxillary premolars were collected from patients who required extraction for an orthodontic reason. The teeth were cleaned and disinfected in 0.1% thymol solution, stored in deionized water in a bottle at 37 °C, and used within 3 months after extraction. The extracted teeth were then examined using a stereomicroscope (SZ 61, Olympus, Japan) at a magnification of 10× to ensure that the teeth were free of cavities, crack lines, restorations, or abnormal tooth surfaces. The recruited teeth were similar in size as determined using a Vernier calliper (Digital Vernier Caliper, Mitutoyo, Japan) and in colour as determined using a spectrophotometer (Vita Easyshade^®^ V, Vitadent, Brea, CA, USA). All measurement was evaluated in the middle one-third of the teeth to calibrate all teeth at A2 shade; the tooth colour was also examined before staining using an Ultrascan Pro (Ultrascan Pro, Hunter Lab, Reston, VA, USA).

### 2.3. Specimen Preparation

The roots of the premolars were cut at the cemento-enamel junction using a low-speed cutting machine (Isomet 1000, Buehler IL, USA). The specimens were placed into a 20 × 12.5 × 9 mm brushing machine mould with the buccal side facing outward. Polyester resin was poured into the mould until 5 mm diameter of the buccal surface emerged from the height of the contour of the teeth. All specimens were stored in deionized water at 37 °C throughout the study.

### 2.4. Staining Procedure

The staining protocol was based on a previous study conducted by Berger SB et al. [3]. All specimens were immersed in red wine (Eaglehawk Merlot, Wolf bass, Australia) for 15 min at room temperature within a pH range of 3 to 4. Then, the specimens were rinsed with distilled water and soaked for 23 h 45 min in artificial saliva (KCl 0.75 gm, MgCl_2_ 0.07 gm, CaCl_2_ 0.199, K_2_HPO_4_ 0.965 gm, KH_2_PO_4_ 0.439 gm, C_8_H_15_NaO_8_ 6 gm, C_6_H_5_COONa 2.4 gm, NaF USP grade 6.6 gm, deionized water 1200 mL) (Faculty of Dentistry, Chulalongkorn University, Bangkok, Thailand) in a temperature-controlled cabinet set to 37 °C [17]. This process was repeated seven days in a row. Then, all specimens were stored in deionized water for the whole study period.

### 2.5. Toothpaste Groups

Specimens were randomly divided into 5 groups. Eight specimens were assigned to each group (Table 1). Surface roughness and colour were initially measured to ensure that there were no significant differences between groups.

### 2.6. Brushing Procedure

Tooth brushing was performed using a brushing simulating machine (V-8 Cross Brushing Machine, SABRI Dentral Enterprises, Villa Park IL, USA) using a soft nylon toothbrush (GUM classic toothbrush soft-4-ROW compact, Sunstar, Singapore). A single toothbrush was used for each specimen and was installed on the machine holder. The brushing machine was set at a frequency of 120 Hz and at a pressure load of 200 g. According to ISO11609:2017, a toothpaste-conditioned medium was prepared by mixing 25 g of toothpaste with 40 mL of deionized water [18]. The brushing procedure was performed for 4 durations: 2 weeks, 1 month, 6 months, and 12 months. According to Vieira-Junior et al., 1 month of brushing is equal to 825 strokes [19]. Therefore, the brushing cycles comprised 412, 825, 4950, and 9900 strokes, respectively. Furthermore, toothbrushes were change every time period of the study. Then, all specimens were cleaned with an ultrasonic cleanser (Ultrasonic cleanser 5210, HEIDOLPH, Germany) and measured for colour and surface roughness after each period. (Period of assessment: 1–15 December 2021.)

### 2.7. Colour Measurement

Colour measurement was assessed after the staining process as a baseline and after each period by spectrophotometer (Ultrascan Pro, Hunter Lab, Reston, VA, USA) with a measurement program (EasyMatch^®^QC, Hunter Lab, Reston, VA, USA). Prior to measurement, the spectrophotometer was calibrated, and the data were collected using a 4 mm reflection port. The specimens were removed from the deionized water, blotted with paper, and measured for colour. The Commission Internationale de l’Eclairage L*a*b* (CIE L*a*b*) colour measurement was repeated three times. Then, the three values were averaged. The difference in colour after brushing was calculated using the CIEDE2000 (ΔE_00_) [20]:

$$\Delta E_{00}=\sqrt{{\left(\frac{\Delta L^{\prime }}{k_{L}S_{L}}\right)}^{2}+{\left(\frac{\Delta C^{\prime }}{k_{C}S_{C}}\right)}^{2}+{\left(\frac{\Delta H^{\prime }}{k_{H}S_{H}}\right)}^{2}+R_{T}\frac{\Delta C^{\prime }}{k_{C}S_{C}}\frac{\Delta H^{\prime }}{k_{H}S_{H}}}$$

### 2.8. Surface Roughness Measurement

The surface roughness of specimens was assessed after the staining process as a baseline and after each period by a non-contact profilometry and measurement program (ALICONA, INFINITEFOCUS SL, Leicestershire, United Kingdom). A surface scan was performed with a 10× magnification lens in the area of 2.0 × 2.0 mm. Three overlapped points around the centre of the specimens were captured. Then, the roughness average (Ra) from each image was calculated and recorded.

### 2.9. Scanning Electron Microscopic Analysis (SEM)

The morphology of toothpaste was investigated using SEM images. Toothpaste-conditioned medium was smeared evenly over the round glass cover, and the sample was properly dried before placing it in a dehumidifier for three days. After desiccation, toothpaste-conditioned medium was coated with gold and captured using a scanning electron microscope (SEM; Quanta 250, FEI, Iowa, United State of America). SEM images were acquired using 20 Kv at magnifications of 1000× and 5000×.

### 2.10. Statistical Analysis

All data were analysed using the statistical program IBM SPSS version 28 (SPSS Armonk, Armonk, NY, USA). For the baseline group, the significance level was set to *p* < 0.05. A Shapiro–Wilk test was applied to evaluate the normality of the data. A one-way analysis of variance (ANOVA) was used to examine differences in surface roughness and colour data among groups at baseline. A significance level of 0.05 was set for all data analysis. A two-way repeated measure analysis of variance (ANOVA) was used to examine the main effects (toothpaste and time) and interaction effects (toothpastes×time) on surface roughness (Ra), ΔE, and ΔL for all time points. The surface roughness, ΔE_00_, and ΔL among all groups were analysed at each time point by one-way ANOVA test with LSD post hoc analysis. Surface roughness, ΔE_00_ and ΔL among all group were analysed at each time point by Friedman test with Dunn’s post hoc analysis. The correlation between surface roughness and ΔE_00_ and between surface roughness and ΔL at each time point were analysed using Spearman’s correlation test.

## 3. Results

### 3.1. Effect of Toothpaste, Time Point, and Interaction on Surface Roughness, ΔE_00_, and ΔL

A two-way repeated ANOVA (Table 2) revealed significant effects of toothpaste (*p* < 0.001) and time *(p* < 0.001) on surface roughness (Ra), ΔE_00_, and ΔL, as well as a significant interaction between these two factors (*p* < 0.001).

### 3.2. Roughness

**Figure 1:**
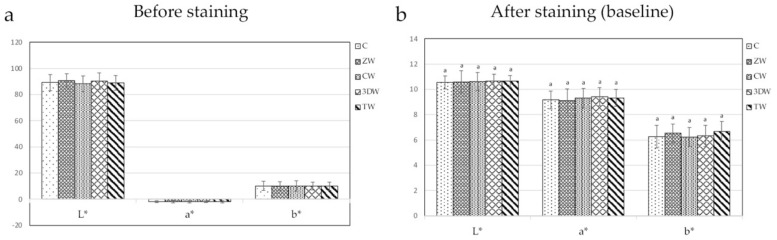
(**a**) Mean L*a*b* value before staining. (**b**) Mean L*a*b* value after staining relative to baseline showed no statistical differences among groups (*p* > 0.05).

**Figure 2:**
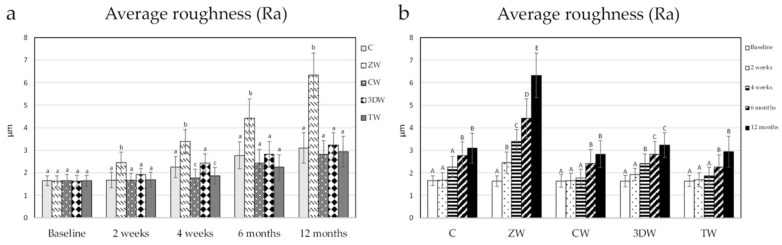
(**a**) Average roughness after brushing at each time point analysed using one-way ANOVA and LSD post hoc test. Different lowercase letters indicate statistically significant differences among toothpaste groups (*p* < 0.05). (**b**) Average roughness after brushing among time points was analysed using Friedman test with Dunn’s post hoc test. Different superscript capital letters indicate statistically significant differences only among the evaluation periods in the same groups (*p* < 0.05).

**Figure 3:**
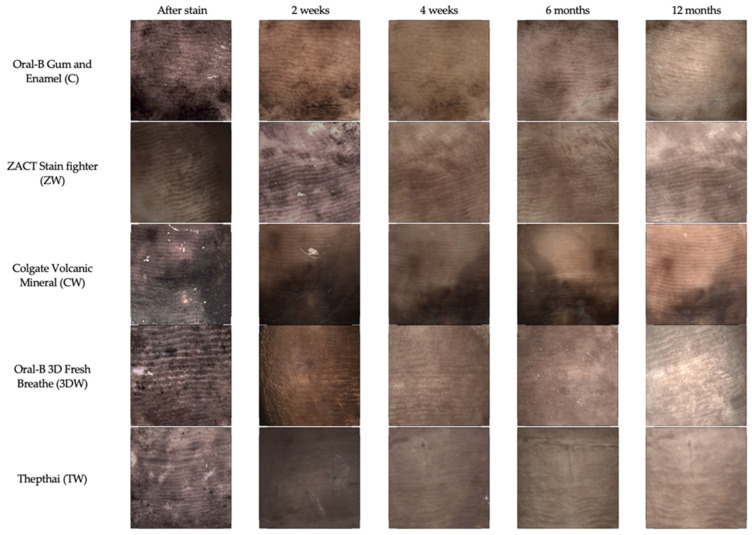
Colour of stained enamel surface of each group after brushing with different toothpastes over time.

All baseline of stained specimens (Ra, L*, a* and b*) showed no statistical difference among groups (*p* = 1.000, *p* = 0.937, *p* = 0.947, *p* = 0.714, respectively) (Figure 1 and Figure 2, Table S1). The result of Ra is shown in Table 3. The ZW group was statistically highest in terms of Ra value compared to other toothpaste group at every time point (2 weeks, *p* = 0.006; 4 weeks, *p* < 0.001; 6 months, *p* < 0.001; 12 months, *p* < 0.001). CW and TW were statistically lowest in terms of roughness among all groups at 4 weeks *(p* < 0.001, *p* < 0.001), but both groups exhibited increased roughness at 6 months, with no significant difference relative to the C and 3DW groups (*p* > 0.05) (Figure 2a, Table S2). After 6 months, there was a significant increase in Ra in the C group (*p* = 0.002). Surface roughness significantly increased between 2 weeks and 12 months in the ZW group (*p* < 0.001). Moreover, surface roughness increased significantly between 4 weeks and 6 months in the 3DW group *(p* = 0.018 and *p* < 0.001,) but did not differ between 6 months and 12 months *(p* = 0.752) (Figure 3b, Table S3).

### 3.3. Colour

**Figure 4:**
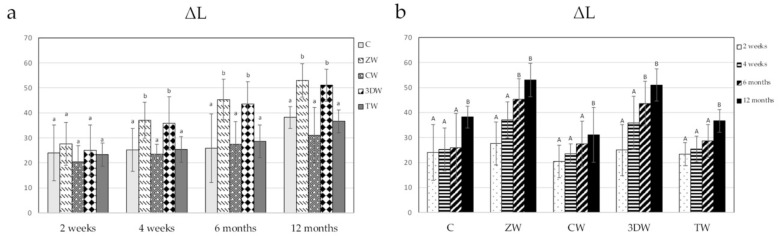
(**a**) ΔL after brushing at each time point analysed using one-way ANOVA and LSD post hoc test. Different lowercase letters indicate statistically significant differences among toothpaste groups (*p* < 0.05). (**b**) ΔL after brushing among time points was analysed using Friedman test with Dunn’s post hoc test. Different capital letters indicate statistically significant differences only among the evaluation periods in the same groups (*p* < 0.05).

**Figure 5:**
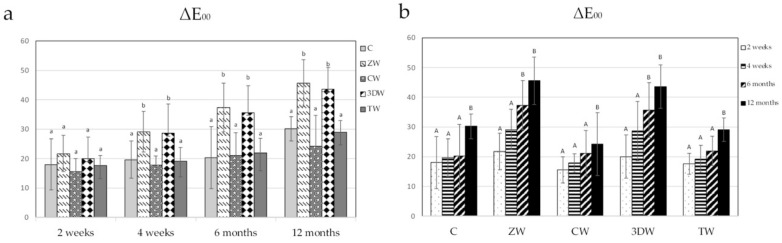
(**a**) ΔE_00_ after brushing at each time point analysed using one-way ANOVA and LSD post hoc test. Different lowercase letters indicate statistically significant differences among toothpaste groups (*p* < 0.05). (**b**) ΔE_00_ after brushing among time points analysed using Friedman test with Dunn’s post hoc test. Different capital letters indicate statistically significant differences only among the evaluation periods in the same groups (*p* < 0.05).

Representative figures of stained tooth surfaces brushed with different toothpaste over time are shown in Figure 3. The mean and standard deviation of ΔL and ΔE_00_ are shown in Table 4 and Table 5, respectively. After 2 weeks, ΔL and ΔE_00_ showed no statistically significant difference among groups (*p* = 0.569). However, after 4 weeks, 6 months, and 12 months, the ΔL and ΔE_00_ values of the ZW and 3DW groups were statistically significantly higher than those of the C, CW, and TW groups (*p* < 0.05) (Figure 4a and Figure 5a, Tables S4 and S6). ΔL and ΔE_00_ were significantly increases in the C, TW, and CW groups after one year of brushing (*p* < 0.05). (Figure 4b and Figure 5b, Tables S5 and S7).

### 3.4. Roughness and Colour Correlation

Correlation coefficient and *p* values are shown in Table 6. A positive significant correlation was observed both between surface roughness and ΔE_00_ and between surface roughness and ΔL at after 4 weeks *(p =* 0.003, *p* = 0.006, *p* = 0.01), indicating that increases in ΔE_00_ and ΔL were influenced by increases in surface roughness. However, after 2 weeks, there were no statistical correlations between surface roughness and ΔE_00_ or between surface roughness and ΔL *(p* = 0.420, *p* = 0.507).

### 3.5. Scanning Electron Microscopic Analysis (SEM)

**Figure 6:**
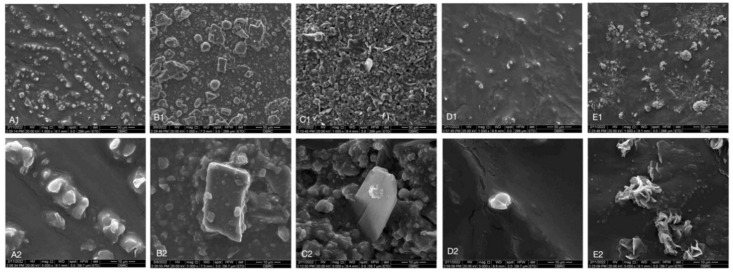
SEM photomicrographs of toothpaste after desiccation. (**A**) Oral-B Gum and Enamel (**A1**) 1000× (**A2**) 5000×; (**B**) ZACT Stain Fighter (**B1**) 1000× (**B2**) 5000×; (**C**) Colgate Optic White Volcanic Mineral (**C1**) 1000× (**C2**) 5000×; (**D**) Oral-B 3D Fresh Breath (**D1**) 1000× (**D2**) 5000×; (**E**) Thepthai (**E1**) 1000× (**E2**) 5000×.

SEM images of each group are shown in Figure 6. The difference in abrasive characteristics was investigated among groups. The ZW group showed the largest rectangular-shaped abrasive particles with a size of 31.28 μm. Moreover, the C and 3DW group showed small, irregular, spherically shaped particles with a size of 8.16 μm and 8.62 μm, respectively. The CW group presented with rectangular-shaped particles with a size of 31.28 μm with a massive volume of small, round particles. The TW group specimens were covered with leaf-like particles with a size of 19.26 μm and a massive amount of small, round particles.

## 4. Discussion

According to a various studies, red wine can cause tooth discolouration more than other methods because its contains chromogenic polyphenols and tannins, which cause dental stains by precipitating on the enamel surface [21,22,23]. Moreover, due to its acidic properties, red wine can demineralize the outer enamel surface, allowing beverage pigments to penetrate the matrices, subsequently causing internalized discolouration [24].

Several studies have reported that the prolonged use of whitening toothpaste can abrade the tooth structure. However, none of these studies evaluated the effect of whitening toothpaste at consecutive time periods for one year [14,15,16,25,26,27]. Hence, it was not specified at which time point whitening toothpaste had the most impact on tooth structure. Therefore, we decided to evaluate both the surface roughness and colour of human enamel specimens at 2 weeks, 4 weeks, 6 months, and 12 months.

All tested toothpastes had an influence on the surface roughness of heavily stained teeth at different periods of time, which can be explained using the Mohs scale of hardness, i.e., “a hard object scratches a soft object” [28]. Enamel has a Mohs scale value of around 5. However, enamel specimens in the present study were immersed in red wine (pH = 3.6), so the outer enamel could be demineralized, resulting reduced hardness. According to our results, ZW contained alumina abrasive particles (Mohs scale = 9.0), increasing the surface roughness of the specimens in two weeks, resulting in the highest surface roughness among all time periods [5]. This result corresponds with a recent study that reported that brushing with alumina-containing toothpaste resulted in higher roughness of enamel than silica-containing toothpaste [29]. CW contained charcoal as an active ingredient, which has a lower Mohs scale of hardness than enamel, so CW toothpaste resulted in less roughness compared with ZW [28]. Previous studies revealed that charcoal particles also slightly increase the average roughness, in agreement with the result of the current study [16,30]. The abrasive agent in C and 3DW was hydrated silica, which has a Mohs scale value equivalent to that of enamel (Mohs = 5); however, both groups showed an increase in surface roughness, which might have been the result of reduced hardness after soaking in acidic solution. However, specimens in the 3DW group did not present with significantly increased surface roughness compared to other groups, suggesting that chemical the agents in the toothpaste did not significantly increase the surface roughness compared to ZW. This finding is consistent with the results of a prior study that compared whitening toothpaste with an antitartar agent to conventional toothpaste with the same abrasive particles. The study revealed that both toothpastes could increase surface roughness, with no significant difference between the groups [8]. As an abrasive agent, TW included calcium carbonate (Mohs hardness = 3.0), which increased surface roughness similarly to other groups, except ZW. Although the Mohs hardness of calcium carbonate is lower than that of enamel, the increase in surface roughness was the result of the reduced hardness of stained enamel, as mentioned above.

The colour change was computed using the CIEDE2000 (E_00_) formula. This formula provides a higher degree of fit, compensating for the nonuniformity of the CIELAB space and for variations in lighting conditions [31]. Colour difference (ΔE) values are perceptible, and colour differences of more than 3.7 are considered clinically unacceptable [32]. In the present study, the colour difference (ΔE) values were divided into three groups: (1) ΔE_00_ less than 0.8 (clinically perceptible), (2) between 0.8 and 1.8 (clinically perceptible but acceptable), and (3) more than 1.8 (not acceptable) [33]. All toothpaste evaluated in the present study demonstrated an unacceptable level of whitening performance after two weeks, with an increase in ΔE_00_ of more than 1.8. A previous in vitro study revealed that alumina-containing toothpaste had the highest capacity to remove stains compared those containing hydrated silica with phosphate chemicals and hydrated silica alone [34]. This result is in agreement with those reported in the present study, since ZW, an alumina-containing toothpaste, and 3DW, a disodium pyrophosphate-containing toothpaste both considerably enhanced the brightness of tooth specimens after four weeks and continued to do so at the end of the test. Moreover, previous research indicated that charcoal can improve the colour of teeth; however, the colour alteration in groups CW and C did not significantly differ, which is consistent with the results of a previous study performed by Vural et al. [14]. The results of the present study demonstrate that the specimens in the TW group did not significantly differ from those in the C and CW groups. A previous study showed that the stain removal ability of calcium carbonate-containing toothpaste is less than that of perlite-containing toothpaste (Mohs hardness = 9) and equal to that of hydrated silica toothpaste [35].

The correlation between the surface roughness and colour alteration was also assessed in this study. Surface roughness—ΔE_00_ and surface roughness—ΔL were significantly correlated after 4 weeks, 6 months, and 12 months. The increase in surface roughness enhances the value of ΔE_00_ and ΔL, as the roughening of the enamel surface can diffuse reflection, resulting in a brightening effect. However, a smooth surface leads to increased specular reflection [36].

The tested toothpastes were inspected under SEM to evaluate the characteristics of their abrasive agents. SEM can be further explained with respect to the effect of the shape and amount of abrasive agents [37]. Large, sharp-edged particles in ZW increased the surface roughness of tooth specimens, whereas the small, round particles in other toothpastes slightly increased surface roughness. In addition, the high particle content of TW and CW may contribute to an increase in surface roughness. In contrast, the low particle content in C and 3DW may have resulted in a less rough surface than ZW.

According to the result of this study, all tested toothpaste increased the brightness of the specimens after 2 weeks. The result showed that 3DW toothpaste slightly changed in surface roughness after brushing for 12 months with no differences in ΔE and ΔL compared with ZW, which resulted in the highest ΔE_00_ and ΔL values after 4 weeks. This result implies that the chemical agent ingredient of whitening toothpaste induced less change in surface morphology with an unacceptable colour alteration.

A limitation of the present study is that the volume loss of tooth structure prior to and following each time period was not quantified. In addition, because the purpose of this study was to simulate a clinical setting, distilled water was excluded from the study. Moreover, the relative dentin abrasiveness (RDA) index of the examined toothpastes should be evaluated further. Because this was an in vitro study, the oral environment could not completely mimic the human oral cavity due to numerous factors, including the saliva factor, which naturally remineralizes the tooth surface tooth, affecting the surface hardness. The presence of the saliva could dilute the concentration of whitening toothpaste, resulting in a reduced effect on the tooth structure. Brushing technique, dietary habits, and oral hygiene habits can also impact surface morphology alterations. Therefore, a clinical study is required for further investigation.

Based on these findings, whitening toothpaste containing chemical agents exhibited the similar whitening effect compared with toothpaste containing hard abrasives. Furthermore, the surface roughness of the tooth samples brushed with toothpaste containing chemical agents showed lower Ra values than those brushed with toothpastes containing hard abrasives. Thus, whitening toothpaste containing chemical agents should be recommended.

## 5. Conclusions

Within the limitations of this in vitro study, it can be concluded that all fives toothpastes caused an increase in surface roughness and colour alteration on red-wine-stained human enamel over time. ZW resulted in the highest surface roughness after 12 months. All tested toothpastes altered the colour of the specimens after 2 weeks. ZW and 3DW resulted in the most considerable changes in colour after 12 months. 3DW demonstrated the greatest effectiveness as a whitening toothpaste because it was less abrasive on stained human enamel and increased the brightness of the specimens over the course of one year. Therefore, patients should be advised to use whitening toothpaste containing chemical agents.

## Figures and Tables

**Table 1:** Brand names, ingredients, manufacturers, abrasive agents, and whitening chemical agents (* conventional toothpaste).

Group, Product Name, and Manufacturer	Ingredients	Abrasive and Whitening Chemical Agents
1. Oral-B Gum and Enamel (C), Procter & Gamble, China * MFG:1121 LOT/EXP:13080386CC/1024	sorbitol, water, sodium lauryl sulphate, flavour, carrageenan, sodium gluconate, stannous chloride, zinc citrate, sodium hydroxide, cellulose gum, sodium saccharin, xanthan gum, calcium aluminium borosilicate, CI77891, CI42090, sodium fluoride (1100 ppm)	Hydrated silica
2. ZACT Stain fighter (ZW), LION, Chonburi, Thailand MFG: 020921 LOT/EXP:005/020924	Water, sorbitol, peg-8, sodium lauryl sulphate, flavour, xanthan gum, sodium benzoate, Chondrus crispus (carrageenan), sodium silicate, sodium saccharin, methylparaben, butylparaben, sodium monofluorophosphate (1100 ppm)	Calcium carbonate, hydrate silica, alumina,
3. Colgate Optic White Volcanic Mineral (CW), Colgate–Palmolive, Chonburi, Thailand MFG: 010621 LOT/EXP: CN123N/010624	Water, sorbitol, volcanic soil, PEG-12, sodium lauryl sulphate, flavour, cellulose gum, potassium hydroxide, phosphoric acid, cocamidopropyl betaine, xanthan gum, sodium saccharin, benzyl alcohol, CI 77019, CI77891, CI77492, CI12085, sodium fluoride (1100 ppm)	Hydrated silica, activated charcoal
4. Oral-B 3D White Luxe Fresh Breath (3DW), Procter & Gamble, China MFG: 0821 LOT/EXP: 12350386CA/0724	Sorbitol, water, hydrated silica *, disodium pyrophosphate, sodium lauryl sulphate, sodium hydroxide, cocamidopropyl betaine, cellulose gum, 5saccharin, carbomer, sodium fluoride (1100 ppm), CI77019, CI77891, CI77492, CI74260	Hydrated silica, disodium pyrophosphate
5. Thepthai (TW), Thepthai Product, Thailand * MFG: 011021 LOT/EXP: E0212102114/011024	Calcium carbonate *, water, sorbitol, sodium lauryl sulphate, sodium carboxymethyl cellulose, borneol, menthol, camphor, Streblus asper leaf extract, guava extract, liquorice extract, clove oil, Eclipta prostrata extract, peppermint extract	Calcium carbonate

**Table 2:** Two-way repeated measures analysis of variance (ANOVA) of toothpaste groups and time on surface roughness, ΔE_00_, and ΔL.

Source	df	Sum of Squares	Mean Square	F	*p*
Surface roughness (Ra)					
Toothpaste group	4	69.16	17.29	22.1	<0.001
Time	4	109.88	27.47	205.2	<0.001
Toothpaste groups ×time	16	42.22	2.64	19.71	<0.001
Error	140	18.74	0.134	-	*-*
ΔE_00_					
Toothpaste group	4	5163.56	1290.90	9.66	<0.001
Time	3	5503.58	1834.53	77.96	<0.001
Toothpaste groups × time	12	1129.23	94.10	4.00	<0.001
Error	105	2470.97	23.53	-	*-*
ΔL					
Toothpaste group	4	6036.43	1509.11	8.93	<0.001
Time	3	6926.50	2308.82	68.23	<0.001
Toothpaste group × time	12	1282.09	106.84	3.157	<0.001
Error	105	3552.98	33.83	-	-

**Table 3:** Mean and standard deviation of roughness (Ra) after brushing. In the same column, different superscript lowercase letters indicate statistically significant differences among toothpaste groups. In the same line, different superscript capital letters indicate statistically significant differences only among the evaluation periods in the same groups (*p* < 0.05).

Toothpaste	Time Point (Ra)				
	Baseline ^1^	2 Weeks ^1^	4 Weeks ^1^	6 Months ^1^	1 Year ^1^
Gum and Enamel (C) ^2^	^a^ 1.65 ± 0.22 ^A^	^a^ 1.67 ± 0.34 ^A^	^a^ 2.25 ± 0.47 ^A^	^a^ 2.77 ± 0.60 ^B^	^a^ 3.10 ± 0.67 ^B^
ZACT Stain Fighter (ZW) ^2^	^a^ 1.63 ± 0.24 ^A^	^b^ 2.45 ± 0.47 ^B^	^b^ 3.39 ± 0.53 ^C^	^b^ 4.42 ± 0.87 ^D^	^b^ 6.33 ± 0.98 ^E^
Colgate Optic Volcanic Mineral (CW) ^2^	^a^ 1.64 ± 0.28 ^A^	^a^ 1.66 ± 0.33 ^A^	^c^ 1.77 ± 0.39 ^A^	^a^ 2.42 ± 0.61 ^B^	^a^ 2.83 ± 0.60 ^B^
Oral-B 3D Fresh Breath (3DW) ^2^	^a^ 1.63 ± 0.25 ^A^	^a^ 1.93 ± 0.27 ^A^	^a^ 2.42 ± 0.42 ^B^	^a^ 2.82 ± 0.57 ^C^	^a^ 3.23 ± 0.55 ^C^
Thepthai (TW) ^2^	^a^ 1.64 ± 0.24 ^A^	^a^ 1.69 ± 0.32 ^A^	^c^ 1.87 ± 0.37 ^A^	^a^ 2.25 ± 0.56 ^B^	^a^ 2.94 ± 0.69 ^B^

^1^ Differences were analysed using one-way ANOVA and LSD post hoc test at each time point. ^2^ Differences were analysed using Friedman test with Dunn’s post hoc test among time points within groups.

**Table 4:** Mean and standard deviation of ΔL (difference between baseline and period of time) after brushing. In the same column, different superscript lowercase letters indicate statistically significant differences among different toothpaste groups. In the same line, different superscript capital letters indicate statistically significant differences only among the evaluation periods in the same groups (*p* < 0.05).

Toothpaste	Time Points (ΔL)			
	Baseline–2 Weeks ^1^	Baseline–4 Weeks ^1^	Baseline–6 Months ^1^	Baseline–12 Months ^1^
Gum and Enamel (C) ^2^	^a^ 24.05 ± 11.16 ^A^	^a^ 25.28 ± 8.6 ^A^	^a^ 25.97 ± 13.70 ^A^	^a^ 38.19 ± 4.34 ^B^
ZACT Stain fighter (ZW) ^2^	^a^ 27.69 ± 8.55 ^A^	^b^ 37.02 ± 7.19 ^B^	^b^ 45.31 ± 8.13 ^B^	^b^ 53.03 ± 6.71 ^B^
Colgate Optic Volcanic Mineral (CW) ^2^	^a^ 20.48 ± 6.53 ^A^	^a^ 23.55 ± 3.99 ^A^	^a^ 27.46 ± 9.09 ^A^	^a^ 31.12 ± 11.00 ^B^
Oral-B 3D Fresh Breath (3DW) ^2^	^a^ 24.99 ± 10.13 ^A^	^b^ 35.82 ± 10.65 ^A^	^b^ 43.61 ± 8.86 ^B^	^b^ 51.03 ± 6.47 ^B^
Thepthai (TW) ^2^	^a^ 23.36 ± 4.64 ^A^	^a^ 25.38 ± 5.12 ^A^	^a^ 28.62 ± 6.52 ^A^	^a^ 36.68 ± 4.53 ^B^

^1^ Differences were analysed using one-way ANOVA and LSD post hoc test at each time point; ^2^ differences were analysed using Friedman test with Dunn’s post hoc test among time points within groups.

**Table 5:** Mean and standard deviation of ΔE_00_ (difference between baseline and period of time) after brushing. In the same column, different superscript lowercase letters indicate statistically significant differences among different toothpaste groups. In the same line, different superscript capital letters indicate statistically significant differences only among the evaluation periods in the same groups (*p* < 0.05).

Toothpastes	Time Points (ΔE_00_)			
	Baseline–2 Weeks ^1^	Baseline–4 Weeks ^1^	Baseline–6 Months ^1^	Baseline–12 Months ^1^
Gum and Enamel (C) ^2^	^a^ 18.02 ± 8.67 ^A^	^a^ 19.64 ± 6.32 ^A^	^a^ 20.30 ± 10.51 ^A^	^a^ 30.17 ± 4.16 ^B^
ZACT Stain fighter (ZW) ^2^	^a^ 21.72 ± 6.15 ^A^	^b^ 29.13 ± 6.85 ^A^	^b^ 37.32 ± 8.30 ^B^	^b^ 45.60 ± 8.01 ^B^
Colgate Optic Volcanic Mineral (CW) ^2^	^a^ 15.60 ± 4.41 ^A^	^a^ 17.87 ± 3.10 ^A^	^a^ 21.13 ± 7.60 ^A^	^a^ 24.25 ± 10.52 ^B^
Oral-B 3D Fresh Breath (3DW) ^2^	^a^ 20.02 ± 7.24 ^A^	^b^ 28.60 ± 9.93 ^A^	^b^ 35.65 ± 9.19 ^B^	^b^ 43.60 ± 7.33 ^B^
Thepthai (TW) ^2^	^a^ 17.63 ± 3.47 ^A^	^a^ 19.21 ± 4.54 ^A^	^a^ 21.9 ± 4.92 ^A^	^a^ 29.00 ± 3.96 ^B^

^1^ Differences were analysed using one-way ANOVA and LSD post hoc test at each time point; ^2^ differences were analysed using Friedman test with Dunn’s post hoc test among time points within groups.

**Table 6:** Spearman’s correlation test was used to assess two pairs at each time point: surface roughness and ΔE_00_, as well as surface roughness and ΔL.

Time		2 Weeks	4 Weeks	6 Months	12 Months
Surface roughness—ΔE_00_	Correlation coefficient	0.131	0.463	0.424	0.402
	*p*-value	0.420	0.003	0.006	0.01
Surface roughness—ΔL	Correlation coefficient	0.108	0.464	0.427	0.402
	*p*-value	0.507	0.003	0.006	0.01

## Data Availability

Not applicable.

## Supplementary Materials

The following supporting information can be downloaded at: https://www.mdpi.com/article/10.3390/dj10100191/s1, Table S1: LSD hoc test showed multiple comparison of L*a*b* baseline among fives toothpaste for baseline.; Table S2: LSD post hoc test showed multiple comparison of surface roughness baseline among fives toothpaste in each time points.; Table S3: Dunn’s post hoc test showed multiple comparison of surface roughness among time points of each toothpaste.; Table S4: LSD post hoc test showed multiple comparison of ΔL among fives toothpaste in each time points.; Table S5: Dunn’s post hoc test showed multiple comparison of ΔL among time points of each toothpaste.; Table S6: LSD post hoc test showed multiple comparison of ΔE_00_ baseline among fives toothpaste in each time points.; Table S7: Dunn’s post hoc test showed multiple comparison of ΔE_00_ among time points of each toothpaste.

## Abbreviations

C	Oral-B Gum and Enamel
ZW	ZACT Stain Fighter
CW	Colgate Optic White Volcanic Mineral
3DW	Oral-B 3D White Luxe Fresh Breath
TW	Thepthai
