# Supplementary material for: Effect of Whitening Toothpaste on Surface Roughness and Colour Alteration of Artificially Extrinsic Stained Human Enamel: In Vitro Study
Source: Dent J (Basel). 2022 Oct 13;10(10):191. doi: 10.3390/dj10100191 (PMC9600486; doi:10.3390/dj10100191)
Supplement: Supplementary file 1 [file dentistry-10-00191-s001.zip › dentistry-1921354-supplementary.pdf]

Effect of Whitening Toothpaste on Surface Roughness and Colour Alteration of Artificially Extrinsic Stained Human Enamel: In Vitro Study

Sarat Suriyasangpetch [1], Pimduean Sivavong [1], Boondarick Niyatiwatchanchai [1], Thanaphum Osathanon [2], Puliwan Gorwong [3], Chawalid Pianmee [4], Dusit Nantanapiboon [1,3,*]

1 Department of Operative Dentistry, Faculty of Dentistry, Chulalongkorn University, Bangkok, 10330 Thailand
2 Dental Stem Cell Biology Research Unit and Department of Anatomy, Faculty of Dentistry, Chulalongkorn University, Bangkok 10330 Thailand
3 Dental Material Research and Development Center, Faculty of Dentistry, Chulalongkorn University, Bangkok, 10330 Thailand
4. Dental Department, Surin Hospital, Surin 32000, Thailand
* Correspondence: dusit.n@chula.ac.th; Tel. +66-2218-8795

Supplementary Tables:

Table S1. LSD hoc test showed multiple comparison of L*a*b* baseline among fives toothpaste for baseline.

Dependent Variable	(I) Toothpaste	(J) Toothpaste	Mean Difference (I-J)	Std. Error	Sig.	95% Confidence Interval	
						Lower Bound	Upper Bound
L* Baseline	Oral B Gum and enamel (C)	ZACT Stain fighter (ZW)	-0.03375	0.32028	0.917	-0.6840	0.6165
		Colgate optic volcano mineral (CW)	-0.04625	0.32028	0.886	-0.6965	0.6040
		Oral B 3D Fresh breathe (3DW)	-0.09250	0.32028	0.774	-0.7427	0.5577
		Thepthai herbal toothpaste (TW)	-0.08375	0.32028	0.795	-0.7340	0.5665
	ZACT Stain fighter (ZW)	Oral B Gum and enamel (C)	0.03375	0.32028	0.917	-0.6165	0.6840
		Colgate optic volcano mineral (CW)	-0.01250	0.32028	0.969	-0.6627	0.6377
		Oral B 3D Fresh breathe (3DW)	-0.05875	0.32028	0.856	-0.7090	0.5915
		Thepthai herbal toothpaste (TW)	-0.05000	0.32028	0.877	-0.7002	0.6002
	Colgate optic volcano mineral (CW)	Oral B Gum and enamel (C)	0.04625	0.32028	0.886	-0.6040	0.6965
		ZACT Stain fighter (ZW)	0.01250	0.32028	0.969	-0.6377	0.6627
		Oral B 3D Fresh breathe (3DW)	-0.04625	0.32028	0.886	-0.6965	0.6040
		Thepthai herbal toothpaste(TW)	-0.03750	0.32028	0.907	-0.6877	0.6127
	Oral B 3D Fresh breathe (3DW)	Oral B Gum and enamel (C)	0.09250	0.32028	0.774	-0.5577	0.7427
		ZACT Stain fighter (ZW)	0.05875	0.32028	0.856	-0.5915	0.7090
		Colgate optic volcano mineral (CW)	0.04625	0.32028	0.886	-0.6040	0.6965
		Thepthai herbal toothpaste (TW)	0.00875	0.32028	0.978	-0.6415	0.6590
	Thepthai herbal toothpaste(TW)	Oral B Gum and enamel (C)	0.08375	0.32028	0.795	-0.5665	0.7340
		ZACT Stain fighter (ZW)	0.05000	0.32028	0.877	-0.6002	0.7002
		Colgate optic volcano mineral (CW)	0.03750	0.32028	0.907	-0.6127	0.6877
		Oral B 3D Fresh breathe (3DW)	-0.00875	0.32028	0.978	-0.6590	0.6415
a* Baseline	Oral B Gum and enamel (C)	ZACT Stain fighter (ZW)	0.05125	0.38226	0.894	-0.7248	0.8273
		Colgate optic volcano mineral (CW)	-0.11750	0.38226	0.760	-0.8935	0.6585
		Oral B 3D Fresh breathe (3DW)	-0.23500	0.38226	0.543	-1.0110	0.5410
		Thepthai herbal toothpaste(TW)	-0.14375	0.38226	0.709	-0.9198	0.6323
	ZACT Stain fighter (ZW)	Oral B Gum and enamel (C)	-0.05125	0.38226	0.894	-0.8273	0.7248
		Colgate optic volcano mineral (CW)	-0.16875	0.38226	0.662	-0.9448	0.6073
		Oral B 3D Fresh breathe (3DW)	-0.28625	0.38226	0.459	-1.0623	0.4898
		Thepthai herbal toothpaste(TW)	-0.19500	0.38226	0.613	-0.9710	0.5810
	Colgate optic volcano mineral (CW)	Oral B Gum and enamel (C)	0.11750	0.38226	0.760	-0.6585	0.8935
		ZACT Stain fighter (ZW)	0.16875	0.38226	0.662	-0.6073	0.9448
		Oral B 3D Fresh breathe (3DW)	-0.11750	0.38226	0.760	-0.8935	0.6585
		Thepthai herbal toothpaste(TW)	-0.02625	0.38226	0.946	-0.8023	0.7498

	Oral B 3D Fresh breathe (3DW)	Oral B Gum and enamel (C)	0.23500	0.38226	0.543	-0.5410	1.0110
		ZACT Stain fighter (ZW)	0.28625	0.38226	0.459	-0.4898	1.0623
		Colgate optic volcano mineral (CW)	0.11750	0.38226	0.760	-0.6585	0.8935
		Thepthai herbal toothpaste(TW)	0.09125	0.38226	0.813	-0.6848	0.8673
	Thepthai herbal toothpaste(TW)	Oral B Gum and enamel (C)	0.14375	0.38226	0.709	-0.6323	0.9198
		ZACT Stain fighter (ZW)	0.19500	0.38226	0.613	-0.5810	0.9710
		Colgate optic volcano mineral (CW)	0.02625	0.38226	0.946	-0.7498	0.8023
		Oral B 3D Fresh breathe (3DW)	-0.09125	0.38226	0.813	-0.8673	0.6848
b* Baseline	Oral B Gum and enamel (C)	ZACT Stain fighter (ZW)	-0.27375	0.40009	0.498	-1.0860	0.5385
		Colgate optic volcano mineral (CW)	0.03250	0.40009	0.936	-0.7797	0.8447
		Oral B 3D Fresh breathe (3DW)	-0.05750	0.40009	0.887	-0.8697	0.7547
		Thepthai herbal toothpaste (TW)	-0.42250	0.40009	0.298	-1.2347	0.3897
	ZACT Stain fighter (ZW)	Oral B Gum and enamel (C)	0.27375	0.40009	0.498	-0.5385	1.0860
		Colgate optic volcano mineral (CW)	0.30625	0.40009	0.449	-0.5060	1.1185
		Oral B 3D Fresh breathe (3DW)	0.21625	0.40009	0.592	-0.5960	1.0285
		Thepthai herbal toothpaste (TW)	-0.14875	0.40009	0.712	-0.9610	0.6635
	Colgate optic volcano mineral (CW)	Oral B Gum and enamel (C)	-0.03250	0.40009	0.936	-0.8447	0.7797
		ZACT Stain fighter (ZW)	-0.30625	0.40009	0.449	-1.1185	0.5060
		Oral B 3D Fresh breathe (3DW)	-0.09000	0.40009	0.823	-0.9022	0.7222
		Thepthai herbal toothpaste (TW)	-0.45500	0.40009	0.263	-1.2672	0.3572
	Oral B 3D Fresh breathe (3DW)	Oral B Gum and enamel (C)	0.05750	0.40009	0.887	-0.7547	0.8697
		ZACT Stain fighter (ZW)	-0.21625	0.40009	0.592	-1.0285	0.5960
		Colgate optic volcano mineral (CW)	0.09000	0.40009	0.823	-0.7222	0.9022
		Thepthai herbal toothpaste (TW)	-0.36500	0.40009	0.368	-1.1772	0.4472
	Thepthai herbal toothpaste(TW)	Oral B Gum and enamel (C)	0.42250	0.40009	0.298	-0.3897	1.2347
		ZACT Stain fighter (ZW)	0.14875	0.40009	0.712	-0.6635	0.9610
		Colgate optic volcano mineral (CW)	0.45500	0.40009	0.263	-0.3572	1.2672
		Oral B 3D Fresh breathe (3DW)	0.36500	0.40009	0.368	-0.4472	1.1772

* The mean difference is significant at the 0.05 level.

Table S2. LSD post hoc test showed multiple comparison of surface roughness baseline among fives toothpaste in each time points.

Dependent Variable	(I) Toothpaste	(J) Toothpaste	Mean Difference (I-J)	Std. Error	Sig.	95% Confidence Interval	
						Lower Bound	Upper Bound
2week	Oral B Gum and enamel (C)	ZACT Stain fighter (ZW)	-3.68000	4.29274	0.397	-12.3947	5.0347
		Colgate optic volcano mineral (CW)	3.53000	4.29274	0.416	-5.1847	12.2447
		Oral B 3D Fresh breathe (3DW)	-1.03750	4.29274	0.810	-9.7522	7.6772
		Thepthai herbal toothpaste(TW)	0.60625	4.29274	0.889	-8.1085	9.3210
	ZACT Stain fighter (ZW)	Oral B Gum and enamel (C)	3.68000	4.29274	0.397	-5.0347	12.3947
		Colgate optic volcano mineral (CW)	7.21000	4.29274	0.102	-1.5047	15.9247
		Oral B 3D Fresh breathe (3DW)	2.64250	4.29274	0.542	-6.0722	11.3572
		Thepthai herbal toothpaste(TW)	4.28625	4.29274	0.325	-4.4285	13.0010
	Colgate optic volcano mineral (CW)	Oral B Gum and enamel (C)	-3.53000	4.29274	0.416	-12.2447	5.1847
		ZACT Stain fighter (ZW)	-7.21000	4.29274	0.102	-15.9247	1.5047
		Oral B 3D Fresh breathe (3DW)	-4.56750	4.29274	0.295	-13.2822	4.1472
		Thepthai herbal toothpaste(TW)	-2.92375	4.29274	0.500	-11.6385	5.7910
	Oral B 3D Fresh breathe (3DW)	Oral B Gum and enamel (C)	1.03750	4.29274	0.810	-7.6772	9.7522
		ZACT Stain fighter (ZW)	-2.64250	4.29274	0.542	-11.3572	6.0722
		Colgate optic volcano mineral (CW)	4.56750	4.29274	0.295	-4.1472	13.2822

		Thepthai herbal toothpaste(TW)	1.64375	4.29274	0.704	-7.0710	10.3585
	Thepthai herbal toothpaste(TW)	Oral B Gum and enamel (C)	-0.60625	4.29274	0.889	-9.3210	8.1085
		ZACT Stain fighter (ZW)	-4.28625	4.29274	0.325	-13.0010	4.4285
		Colgate optic volcano mineral (CW)	2.92375	4.29274	0.500	-5.7910	11.6385
		Oral B 3D Fresh breathe (3DW)	-1.64375	4.29274	0.704	-10.3585	7.0710
4weeks	Oral B Gum and enamel (C)	ZACT Stain fighter (ZW)	-10.60125*	4.58953	0.027	-19.9185	-1.2840
		Colgate optic volcano mineral (CW)	1.68500	4.58953	0.716	-7.6322	11.0022
		Oral B 3D Fresh breathe (3DW)	-7.72250	4.58953	0.101	-17.0397	1.5947
		Thepthai herbal toothpaste(TW)	-4.09750	4.58953	0.378	-13.4147	5.2197
	ZACT Stain fighter (ZW)	Oral B Gum and enamel (C)	10.60125*	4.58953	0.027	1.2840	19.9185
		Colgate optic volcano mineral (CW)	12.28625*	4.58953	0.011	2.9690	21.6035
		Oral B 3D Fresh breathe (3DW)	2.87875	4.58953	0.535	-6.4385	12.1960
		Thepthai herbal toothpaste(TW)	6.50375	4.58953	0.165	-2.8135	15.8210
	Colgate optic volcano mineral (CW)	Oral B Gum and enamel (C)	-1.68500	4.58953	0.716	-11.0022	7.6322
		ZACT Stain fighter (ZW)	-12.28625*	4.58953	0.011	-21.6035	-2.9690
		Oral B 3D Fresh breathe (3DW)	-9.40750*	4.58953	0.048	-18.7247	-0.0903
		Thepthai herbal toothpaste(TW)	-5.78250	4.58953	0.216	-15.0997	3.5347
	Oral B 3D Fresh breathe (3DW)	Oral B Gum and enamel (C)	7.72250	4.58953	0.101	-1.5947	17.0397
		ZACT Stain fighter (ZW)	-2.87875	4.58953	0.535	-12.1960	6.4385
		Colgate optic volcano mineral (CW)	9.40750*	4.58953	0.048	0.0903	18.7247
		Thepthai herbal toothpaste(TW)	3.62500	4.58953	0.435	-5.6922	12.9422
	Thepthai herbal toothpaste(TW)	Oral B Gum and enamel (C)	4.09750	4.58953	0.378	-5.2197	13.4147
		ZACT Stain fighter (ZW)	-6.50375	4.58953	0.165	-15.8210	2.8135
		Colgate optic volcano mineral (CW)	5.78250	4.58953	0.216	-3.5347	15.0997
		Oral B 3D Fresh breathe (3DW)	-3.62500	4.58953	0.435	-12.9422	5.6922
6 months	Oral B Gum and enamel (C)	ZACT Stain fighter (ZW)	-16.75875*	5.66938	0.006	-28.2682	-5.2493
		Colgate optic volcano mineral (CW)	-1.54250	5.66938	0.787	-13.0520	9.9670
		Oral B 3D Fresh breathe (3DW)	-14.58500*	5.66938	0.014	-26.0945	-3.0755
		Thepthai herbal toothpaste(TW)	-5.66500	5.66938	0.325	-17.1745	5.8445
	ZACT Stain fighter (ZW)	Oral B Gum and enamel (C)	16.75875*	5.66938	0.006	5.2493	28.2682
		Colgate optic volcano mineral (CW)	15.21625*	5.66938	0.011	3.7068	26.7257
		Oral B 3D Fresh breathe (3DW)	2.17375	5.66938	0.704	-9.3357	13.6832
		Thepthai herbal toothpaste(TW)	11.09375	5.66938	0.058	-0.4157	22.6032
	Colgate optic volcano mineral (CW)	Oral B Gum and enamel (C)	1.54250	5.66938	0.787	-9.9670	13.0520
		ZACT Stain fighter (ZW)	-15.21625*	5.66938	0.011	-26.7257	-3.7068
		Oral B 3D Fresh breathe (3DW)	-13.04250*	5.66938	0.027	-24.5520	-1.5330
		Thepthai herbal toothpaste(TW)	-4.12250	5.66938	0.472	-15.6320	7.3870
	Oral B 3D Fresh breathe (3DW)	Oral B Gum and enamel (C)	14.58500*	5.66938	0.014	3.0755	26.0945
		ZACT Stain fighter (ZW)	-2.17375	5.66938	0.704	-13.6832	9.3357
		Colgate optic volcano mineral (CW)	13.04250*	5.66938	0.027	1.5330	24.5520
		Thepthai herbal toothpaste(TW)	8.92000	5.66938	0.125	-2.5895	20.4295

	Thepthai herbal toothpaste(TW)	Oral B Gum and enamel (C)	5.66500	5.66938	0.325	-5.8445	17.1745
		ZACT Stain fighter (ZW)	-11.09375	5.66938	0.058	-22.6032	0.4157
		Colgate optic volcano mineral (CW)	4.12250	5.66938	0.472	-7.3870	15.6320
		Oral B 3D Fresh breathe (3DW)	-8.92000	5.66938	0.125	-20.4295	2.5895
12 months	Oral B Gum and enamel (C)	ZACT Stain fighter (ZW)	-14.09875*	3.70849	0.001	-21.6274	-6.5701
		Colgate optic volcano mineral (CW)	7.02125	3.70849	0.067	-0.5074	14.5499
		Oral B 3D Fresh breathe (3DW)	-11.98625*	3.70849	0.003	-19.5149	-4.4576
		Thepthai herbal toothpaste(TW)	1.41750	3.70849	0.705	-6.1111	8.9461
	ZACT Stain fighter (ZW)	Oral B Gum and enamel (C)	14.09875*	3.70849	0.001	6.5701	21.6274
		Colgate optic volcano mineral (CW)	21.12000*	3.70849	0.000	13.5914	28.6486
		Oral B 3D Fresh breathe (3DW)	2.11250	3.70849	0.573	-5.4161	9.6411
		Thepthai herbal toothpaste(TW)	15.51625*	3.70849	0.000	7.9876	23.0449
	Colgate optic volcano mineral (CW)	Oral B Gum and enamel (C)	-7.02125	3.70849	0.067	-14.5499	0.5074
		ZACT Stain fighter (ZW)	-21.12000*	3.70849	0.000	-28.6486	-13.5914
		Oral B 3D Fresh breathe (3DW)	-19.00750*	3.70849	0.000	-26.5361	-11.4789
		Thepthai herbal toothpaste(TW)	-5.60375	3.70849	0.140	-13.1324	1.9249
	Oral B 3D Fresh breathe (3DW)	Oral B Gum and enamel (C)	11.98625*	3.70849	0.003	4.4576	19.5149
		ZACT Stain fighter (ZW)	-2.11250	3.70849	0.573	-9.6411	5.4161
		Colgate optic volcano mineral (CW)	19.00750*	3.70849	0.000	11.4789	26.5361
		Thepthai herbal toothpaste(TW)	13.40375*	3.70849	0.001	5.8751	20.9324
	Thepthai herbal toothpaste(TW)	Oral B Gum and enamel (C)	-1.41750	3.70849	0.705	-8.9461	6.1111
		ZACT Stain fighter (ZW)	-15.51625*	3.70849	0.000	-23.0449	-7.9876
		Colgate optic volcano mineral (CW)	5.60375	3.70849	0.140	-1.9249	13.1324
		Oral B 3D Fresh breathe (3DW)	-13.40375*	3.70849	0.001	-20.9324	-5.8751

*. The mean difference is significant at the 0.05 level.

Table S3. Dunn's post hoc test showed multiple comparison of surface roughness among time points of each toothpaste.

Toothpastes	Time points	Test Statistic	Std. Error	Std. Test Statistic	Sig.
Oral-B gum and enamel (C)	Baseline - 2 weeks	-0.375	0.791	-0.474	0.635
	Baseline - 4 weeks	-1.375	0.791	-1.739	0.082
	Baseline - 6 months	-2.500	0.791	-3.162	0.002
	Baseline - 12 months	-3.250	0.791	-4.111	0.000
	2 weeks - 4 weeks	-1.000	0.791	-1.265	0.206
	2 weeks - 6 months	-2.125	0.791	-2.688	0.007
	2 weeks - 12 months	-2.875	0.791	-3.637	0.000
	4 weeks - 6 months	-1.125	0.791	-1.423	0.155
	4 weeks - 12 months	-1.875	0.791	-2.372	0.018
	6 months - 12 months	-0.750	0.791	-0.949	0.343
ZACT Staing fighter (ZW)	Baseline - 2 weeks	-1.000	0.791	-1.265	0.206
	Baseline - 4 weeks	-2.000	0.791	-2.530	0.011

	Baseline - 6 months	-3.000	0.791	-3.795	0.000
	Baseline - 12 months	-4.000	0.791	-5.060	0.000
	2 weeks - 4 weeks	-1.000	0.791	-1.265	0.206
	2 weeks - 6 months	-2.000	0.791	-2.530	0.011
	2 weeks - 12 months	-3.000	0.791	-3.795	0.000
	4 weeks - 6 months	-1.000	0.791	-1.265	0.206
	4 weeks - 12 months	-2.000	0.791	-2.530	0.011
	6 months - 12 months	-1.000	0.791	-1.265	0.206
Colgate optic volcanic mineral (CW)	Baseline - 2 weeks	-0.125	0.791	-0.158	0.874
	Baseline - 4 weeks	-0.875	0.791	-1.107	0.268
	Baseline - 6 months	-2.125	0.791	-2.688	0.007
	Baseline - 12 months	-3.125	0.791	-3.953	0.000
	2 weeks - 4 weeks	-0.750	0.791	-0.949	0.343
	2 weeks - 6 months	-2.000	0.791	-2.530	0.011
	2 weeks - 12 months	-3.000	0.791	-3.795	0.000
	4 weeks - 6 months	-1.250	0.791	-1.581	0.114
	4 weeks - 12 months	-2.250	0.791	-2.846	0.004
	6 months - 12 months	-1.000	0.791	-1.265	0.206
Oral B 3D Fresh breath	Baseline - 2 weeks	-0.750	0.791	-0.949	0.343
	Baseline - 4 weeks	-1.875	0.791	-2.372	0.018
	Baseline - 6 months	-3.250	0.791	-4.111	0.000
	Baseline - 12 months	-3.500	0.791	-4.427	0.000
	2 weeks - 4 weeks	-1.125	0.791	-1.423	0.155
	2 weeks - 6 months	-2.500	0.791	-3.162	0.002
	2 weeks - 12 months	-2.750	0.791	-3.479	0.001
	4 weeks - 6 months	-1.375	0.791	-1.739	0.082
	4 weeks - 12 months	-1.625	0.791	-2.055	0.040
	6 months - 12 months	-0.250	0.791	-0.316	0.752
Thepthai (TW)	Baseline - 2 weeks	-0.125	0.791	-0.158	0.874
	Baseline - 4 weeks	-1.063	0.791	-1.344	0.179
	Baseline - 6 months	-2.313	0.791	-2.925	0.003
	Baseline - 12 months	-3.375	0.791	-4.269	0.000
	2 weeks - 4 weeks	-0.938	0.791	-1.186	0.236
	2 weeks - 6 months	-2.188	0.791	-2.767	0.006
	2 weeks - 12 months	-3.250	0.791	-4.111	0.000
	4 weeks - 6 months	-1.250	0.791	-1.581	0.114
	4 weeks - 12 months	-2.313	0.791	-2.925	0.003
	6 months - 12 months	-1.063	0.791	-1.344	0.179

Table S4. LSD post hoc test showed multiple comparison of ΔL among fives toothpaste in each time points.

Dependent Variable	(I) Toothpaste	(J) Toothpaste	Mean Difference (I-J)	Std. Error	Sig.	95% Confidence Interval	
						Lower Bound	Upper Bound
2 weeks	Oral B Gum and enamel (C)	ZACT Stain fighter (ZW)	-3.68000	4.29274	0.397	-12.3947	5.0347
		Colgate optic volcano mineral (CW)	3.53000	4.29274	0.416	-5.1847	12.2447
		Oral B 3D Fresh breathe (3DW)	-1.03750	4.29274	0.810	-9.7522	7.6772
		Thepthai herbal toothpaste(TW)	0.60625	4.29274	0.889	-8.1085	9.3210
	ZACT Stain fighter (ZW)	Oral B Gum and enamel (C)	3.68000	4.29274	0.397	-5.0347	12.3947
		Colgate optic volcano mineral (CW)	7.21000	4.29274	0.102	-1.5047	15.9247
		Oral B 3D Fresh breathe (3DW)	2.64250	4.29274	0.542	-6.0722	11.3572
		Thepthai herbal toothpaste(TW)	4.28625	4.29274	0.325	-4.4285	13.0010
	Colgate optic volcano mineral (CW)	Oral B Gum and enamel (C)	-3.53000	4.29274	0.416	-12.2447	5.1847
		ZACT Stain fighter (ZW)	-7.21000	4.29274	0.102	-15.9247	1.5047
		Oral B 3D Fresh breathe (3DW)	-4.56750	4.29274	0.295	-13.2822	4.1472
		Thepthai herbal toothpaste(TW)	-2.92375	4.29274	0.500	-11.6385	5.7910
	Oral B 3D Fresh breathe (3DW)	Oral B Gum and enamel (C)	1.03750	4.29274	0.810	-7.6772	9.7522
		ZACT Stain fighter (ZW)	-2.64250	4.29274	0.542	-11.3572	6.0722
		Colgate optic volcano mineral (CW)	4.56750	4.29274	0.295	-4.1472	13.2822
		Thepthai herbal toothpaste(TW)	1.64375	4.29274	0.704	-7.0710	10.3585
	Thepthai herbal toothpaste(TW)	Oral B Gum and enamel (C)	-0.60625	4.29274	0.889	-9.3210	8.1085
		ZACT Stain fighter (ZW)	-4.28625	4.29274	0.325	-13.0010	4.4285
		Colgate optic volcano mineral (CW)	2.92375	4.29274	0.500	-5.7910	11.6385
		Oral B 3D Fresh breathe (3DW)	-1.64375	4.29274	0.704	-10.3585	7.0710
4 weeks	Oral B Gum and enamel (C)	ZACT Stain fighter (ZW)	-10.60125*	4.58953	0.027	-19.9185	-1.2840
		Colgate optic volcano mineral (CW)	1.68500	4.58953	0.716	-7.6322	11.0022
		Oral B 3D Fresh breathe (3DW)	-7.72250	4.58953	0.101	-17.0397	1.5947
		Thepthai herbal toothpaste(TW)	-4.09750	4.58953	0.378	-13.4147	5.2197
	ZACT Stain fighter (ZW)	Oral B Gum and enamel (C)	10.60125*	4.58953	0.027	1.2840	19.9185
		Colgate optic volcano mineral (CW)	12.28625*	4.58953	0.011	2.9690	21.6035
		Oral B 3D Fresh breathe (3DW)	2.87875	4.58953	0.535	-6.4385	12.1960
		Thepthai herbal toothpaste(TW)	6.50375	4.58953	0.165	-2.8135	15.8210
	Colgate optic volcano mineral (CW)	Oral B Gum and enamel (C)	-1.68500	4.58953	0.716	-11.0022	7.6322
		ZACT Stain fighter (ZW)	-12.28625*	4.58953	0.011	-21.6035	-2.9690
		Oral B 3D Fresh breathe (3DW)	-9.40750*	4.58953	0.048	-18.7247	-0.0903
		Thepthai herbal toothpaste(TW)	-5.78250	4.58953	0.216	-15.0997	3.5347
	Oral B 3D Fresh breathe (3DW)	Oral B Gum and enamel (C)	7.72250	4.58953	0.101	-1.5947	17.0397
		ZACT Stain fighter (ZW)	-2.87875	4.58953	0.535	-12.1960	6.4385
		Colgate optic volcano mineral (CW)	9.40750*	4.58953	0.048	0.0903	18.7247
		Thepthai herbal toothpaste(TW)	3.62500	4.58953	0.435	-5.6922	12.9422
	Thepthai herbal toothpaste(TW)	Oral B Gum and enamel (C)	4.09750	4.58953	0.378	-5.2197	13.4147
		ZACT Stain fighter (ZW)	-6.50375	4.58953	0.165	-15.8210	2.8135
		Colgate optic volcano mineral (CW)	5.78250	4.58953	0.216	-3.5347	15.0997
		Oral B 3D Fresh breathe (3DW)	-3.62500	4.58953	0.435	-12.9422	5.6922
6 months	Oral B Gum and enamel (C)	ZACT Stain fighter (ZW)	-16.75875*	5.66938	0.006	-28.2682	-5.2493
		Colgate optic volcano mineral (CW)	-1.54250	5.66938	0.787	-13.0520	9.9670
		Oral B 3D Fresh breathe (3DW)	-14.58500*	5.66938	0.014	-26.0945	-3.0755
		Thepthai herbal toothpaste(TW)	-5.66500	5.66938	0.325	-17.1745	5.8445
	ZACT Stain fighter (ZW)	Oral B Gum and enamel (C)	16.75875*	5.66938	0.006	5.2493	28.2682
		Colgate optic volcano mineral (CW)	15.21625*	5.66938	0.011	3.7068	26.7257
		Oral B 3D Fresh breathe (3DW)	2.17375	5.66938	0.704	-9.3357	13.6832
		Thepthai herbal toothpaste(TW)	11.09375	5.66938	0.058	-0.4157	22.6032
	Colgate optic volcano mineral (CW)	Oral B Gum and enamel (C)	1.54250	5.66938	0.787	-9.9670	13.0520
		ZACT Stain fighter (ZW)	-15.21625*	5.66938	0.011	-26.7257	-3.7068
		Oral B 3D Fresh breathe (3DW)	-13.04250*	5.66938	0.027	-24.5520	-1.5330

		Thepthai herbal toothpaste(TW)	-4.12250	5.66938	0.472	-15.6320	7.3870
	Oral B 3D Fresh breathe (3DW)	Oral B Gum and enamel (C)	14.58500*	5.66938	0.014	3.0755	26.0945
		ZACT Stain fighter (ZW)	-2.17375	5.66938	0.704	-13.6832	9.3357
		Colgate optic volcano mineral (CW)	13.04250*	5.66938	0.027	1.5330	24.5520
		Thepthai herbal toothpaste(TW)	8.92000	5.66938	0.125	-2.5895	20.4295
	Thepthai herbal toothpaste(TW)	Oral B Gum and enamel (C)	5.66500	5.66938	0.325	-5.8445	17.1745
		ZACT Stain fighter (ZW)	-11.09375	5.66938	0.058	-22.6032	0.4157
		Colgate optic volcano mineral (CW)	4.12250	5.66938	0.472	-7.3870	15.6320
		Oral B 3D Fresh breathe (3DW)	-8.92000	5.66938	0.125	-20.4295	2.5895
12 months	Oral B Gum and enamel (C)	ZACT Stain fighter (ZW)	-14.09875*	3.70849	0.001	-21.6274	-6.5701
		Colgate optic volcano mineral (CW)	7.02125	3.70849	0.067	-0.5074	14.5499
		Oral B 3D Fresh breathe (3DW)	-11.98625*	3.70849	0.003	-19.5149	-4.4576
		Thepthai herbal toothpaste(TW)	1.41750	3.70849	0.705	-6.1111	8.9461
	ZACT Stain fighter (ZW)	Oral B Gum and enamel (C)	14.09875*	3.70849	0.001	6.5701	21.6274
		Colgate optic volcano mineral (CW)	21.12000*	3.70849	0.000	13.5914	28.6486
		Oral B 3D Fresh breathe (3DW)	2.11250	3.70849	0.573	-5.4161	9.6411
		Thepthai herbal toothpaste(TW)	15.51625*	3.70849	0.000	7.9876	23.0449
	Colgate optic volcano mineral (CW)	Oral B Gum and enamel (C)	-7.02125	3.70849	0.067	-14.5499	0.5074
		ZACT Stain fighter (ZW)	-21.12000*	3.70849	0.000	-28.6486	-13.5914
		Oral B 3D Fresh breathe (3DW)	-19.00750*	3.70849	0.000	-26.5361	-11.4789
		Thepthai herbal toothpaste(TW)	-5.60375	3.70849	0.140	-13.1324	1.9249
	Oral B 3D Fresh breathe (3DW)	Oral B Gum and enamel (C)	11.98625*	3.70849	0.003	4.4576	19.5149
		ZACT Stain fighter (ZW)	-2.11250	3.70849	0.573	-9.6411	5.4161
		Colgate optic volcano mineral (CW)	19.00750*	3.70849	0.000	11.4789	26.5361
		Thepthai herbal toothpaste(TW)	13.40375*	3.70849	0.001	5.8751	20.9324
	Thepthai herbal toothpaste(TW)	Oral B Gum and enamel (C)	-1.41750	3.70849	0.705	-8.9461	6.1111
		ZACT Stain fighter (ZW)	-15.51625*	3.70849	0.000	-23.0449	-7.9876
		Colgate optic volcano mineral (CW)	5.60375	3.70849	0.140	-1.9249	13.1324
		Oral B 3D Fresh breathe (3DW)	-13.40375*	3.70849	0.001	-20.9324	-5.8751

* The mean difference is significant at the 0.05 level.

Table S5. Dunn's post hoc test showed multiple comparison of ΔL among time points of each toothpaste.

Toothpastes	Time points	Test Statistic	Std. Error	Std. Test Statistic	Sig.
Oral-B gum and enamel (C)	2 weeks - 4 weeks	-0.500	0.645	-0.775	0.439
	2 weeks - 6 months	-0.500	0.645	-0.775	0.439
	2 weeks - 12 months	-2.000	0.645	-3.098	0.002
	4 weeks - 6 months	0.000	0.645	0.000	1.000
	4 weeks - 12 months	-1.500	0.645	-2.324	0.020
	6 months - 12 months	-1.500	0.645	-2.324	0.020
ZACT Staing fighter (ZW)	2 weeks - 4 weeks	-0.750	0.645	-1.162	0.245
	2 weeks - 6 months	-1.875	0.645	-2.905	0.004
	2 weeks - 12 months	-2.875	0.645	-4.454	0.000
	4 weeks - 6 months	-1.125	0.645	-1.743	0.081
	4 weeks - 12 months	-2.125	0.645	-3.292	0.001
	6 months - 12 months	-1.000	0.645	-1.549	0.121
Colgate optic volcanic mineral (CW)	2 weeks - 4 weeks	-0.250	0.645	-0.387	0.699
	2 weeks - 6 months	-1.125	0.645	-1.743	0.081
	2 weeks - 12 months	-1.625	0.645	-2.517	0.012

	4 weeks - 6 months	-0.875	0.645	-1.356	0.175
	4 weeks - 12 months	-1.375	0.645	-2.130	0.033
	6 months - 12 months	-0.500	0.645	-0.775	0.439
Oral B 3D Fresh breath (3DW)	2 weeks - 4 weeks	-1.000	0.645	-1.549	0.121
	2 weeks - 6 months	-2.125	0.645	-3.292	0.001
	2 weeks - 12 months	-2.875	0.645	-4.454	0.000
	4 weeks - 6 months	-1.125	0.645	-1.743	0.081
	4 weeks - 12 months	-1.875	0.645	-2.905	0.004
	6 months - 12 months	-0.750	0.645	-1.162	0.245
Thepthai (TW)	2 weeks - 6 months	-0.375	0.645	-0.581	0.561
	2 weeks - 4 weeks	-0.500	0.645	-0.775	0.439
	2 weeks - 12 months	-2.125	0.645	-3.292	0.001
	6 months - 4 weeks	0.125	0.645	0.194	0.846
	6 months - 12 months	-1.750	0.645	-2.711	0.007
	4 weeks - 12 months	-1.625	0.645	-2.517	0.012

Table S6. LSD post hoc test showed multiple comparison of ΔE_{00} baseline among fives toothpaste in each time points.

Dependent Variable	(I) Toothpaste	(J) Toothpaste	Mean Difference (I-J)	Std. Error	Sig.	95% Confidence Interval	
						Lower Bound	Upper Bound
2 weeks	Oral B Gum and enamel (C)	ZACT Stain fighter (ZW)	-4.28067	3.92897	0.283	-12.2569	3.6956
		Colgate optic volcano mineral (CW)	2.76525	3.92897	0.486	-5.2110	10.7415
		Oral B 3D Fresh breathe (3DW)	-1.88778	3.92897	0.634	-9.8640	6.0884
		Thepthai herbal toothpaste(TW)	0.32772	3.92897	0.934	-7.6485	8.3039
	ZACT Stain fighter (ZW)	Oral B Gum and enamel (C)	4.28067	3.92897	0.283	-3.6956	12.2569
		Colgate optic volcano mineral (CW)	7.04592	3.92897	0.082	-0.9303	15.0221
		Oral B 3D Fresh breathe (3DW)	2.39289	3.92897	0.546	-5.5833	10.3691
		Thepthai herbal toothpaste(TW)	4.60839	3.92897	0.249	-3.3678	12.5846
	Colgate optic volcano mineral (CW)	Oral B Gum and enamel (C)	-2.76525	3.92897	0.486	-10.7415	5.2110
		ZACT Stain fighter (ZW)	-7.04592	3.92897	0.082	-15.0221	0.9303
		Oral B 3D Fresh breathe (3DW)	-4.65303	3.92897	0.244	-12.6293	3.3232
		Thepthai herbal toothpaste(TW)	-2.43753	3.92897	0.539	-10.4138	5.5387
	Oral B 3D Fresh breathe (3DW)	Oral B Gum and enamel (C)	1.88778	3.92897	0.634	-6.0884	9.8640
		ZACT Stain fighter (ZW)	-2.39289	3.92897	0.546	-10.3691	5.5833
		Colgate optic volcano mineral (CW)	4.65303	3.92897	0.244	-3.3232	12.6293
		Thepthai herbal toothpaste(TW)	2.21550	3.92897	0.576	-5.7607	10.1917
	Thepthai herbal toothpaste(TW)	Oral B Gum and enamel (C)	-0.32772	3.92897	0.934	-8.3039	7.6485
		ZACT Stain fighter (ZW)	-4.60839	3.92897	0.249	-12.5846	3.3678
		Colgate optic volcano mineral (CW)	2.43753	3.92897	0.539	-5.5387	10.4138
		Oral B 3D Fresh breathe (3DW)	-2.21550	3.92897	0.576	-10.1917	5.7607
4 weeks	Oral B Gum and enamel (C)	ZACT Stain fighter (ZW)	-11.23073*	3.66737	0.004	-18.6759	-3.7856
		Colgate optic volcano mineral (CW)	1.89672	3.66737	0.608	-5.5484	9.3419
		Oral B 3D Fresh breathe (3DW)	-10.28617*	3.66737	0.008	-17.7313	-2.8410
		Thepthai herbal toothpaste(TW)	0.17268	3.66737	0.963	-7.2725	7.6178
	ZACT Stain fighter (ZW)	Oral B Gum and enamel (C)	11.23073*	3.66737	0.004	3.7856	18.6759
		Colgate optic volcano mineral (CW)	13.12744*	3.66737	0.001	5.6823	20.5726
		Oral B 3D Fresh breathe (3DW)	0.94456	3.66737	0.798	-6.5006	8.3897
		Thepthai herbal toothpaste(TW)	11.40341*	3.66737	0.004	3.9583	18.8486
		Oral B Gum and enamel (C)	-1.89672	3.66737	0.608	-9.3419	5.5484

	Colgate optic volcano mineral (CW)	ZACT Stain fighter (ZW)	-13.12744*	3.66737	0.001	-20.5726	-5.6823
		Oral B 3D Fresh breathe (3DW)	-12.18288*	3.66737	0.002	-19.6280	-4.7377
		Thepthai herbal toothpaste(TW)	-1.72403	3.66737	0.641	-9.1692	5.7211
	Oral B 3D Fresh breathe (3DW)	Oral B Gum and enamel (C)	10.28617*	3.66737	0.008	2.8410	17.7313
		ZACT Stain fighter (ZW)	-0.94456	3.66737	0.798	-8.3897	6.5006
		Colgate optic volcano mineral (CW)	12.18288*	3.66737	0.002	4.7377	19.6280
		Thepthai herbal toothpaste(TW)	10.45885*	3.66737	0.007	3.0137	17.9040
	Thepthai herbal toothpaste(TW)	Oral B Gum and enamel (C)	-0.17268	3.66737	0.963	-7.6178	7.2725
		ZACT Stain fighter (ZW)	-11.40341*	3.66737	0.004	-18.8486	-3.9583
		Colgate optic volcano mineral (CW)	1.72403	3.66737	0.641	-5.7211	9.1692
		Oral B 3D Fresh breathe (3DW)	-10.45885*	3.66737	0.007	-17.9040	-3.0137
6 months	Oral B Gum and enamel (C)	ZACT Stain fighter (ZW)	-18.79640*	4.62302	0.000	-28.1816	-9.4112
		Colgate optic volcano mineral (CW)	-1.48728	4.62302	0.750	-10.8725	7.8980
		Oral B 3D Fresh breathe (3DW)	-17.15408*	4.62302	0.001	-26.5393	-7.7688
		Thepthai herbal toothpaste(TW)	-2.38810	4.62302	0.609	-11.7733	6.9971
	ZACT Stain fighter (ZW)	Oral B Gum and enamel (C)	18.79640*	4.62302	0.000	9.4112	28.1816
		Colgate optic volcano mineral (CW)	17.30911*	4.62302	0.001	7.9239	26.6943
		Oral B 3D Fresh breathe (3DW)	1.64231	4.62302	0.725	-7.7429	11.0275
		Thepthai herbal toothpaste(TW)	16.40829*	4.62302	0.001	7.0231	25.7935
	Colgate optic volcano mineral (CW)	Oral B Gum and enamel (C)	1.48728	4.62302	0.750	-7.8980	10.8725
		ZACT Stain fighter (ZW)	-17.30911*	4.62302	0.001	-26.6943	-7.9239
		Oral B 3D Fresh breathe (3DW)	-15.66680*	4.62302	0.002	-25.0520	-6.2816
		Thepthai herbal toothpaste(TW)	-0.90082	4.62302	0.847	-10.2861	8.4844
	Oral B 3D Fresh breathe (3DW)	Oral B Gum and enamel (C)	17.15408*	4.62302	0.001	7.7688	26.5393
		ZACT Stain fighter (ZW)	-1.64231	4.62302	0.725	-11.0275	7.7429
		Colgate optic volcano mineral (CW)	15.66680*	4.62302	0.002	6.2816	25.0520
		Thepthai herbal toothpaste(TW)	14.76598*	4.62302	0.003	5.3807	24.1512
	Thepthai herbal toothpaste(TW)	Oral B Gum and enamel (C)	2.38810	4.62302	0.609	-6.9971	11.7733
		ZACT Stain fighter (ZW)	-16.40829*	4.62302	0.001	-25.7935	-7.0231
		Colgate optic volcano mineral (CW)	0.90082	4.62302	0.847	-8.4844	10.2861
		Oral B 3D Fresh breathe (3DW)	-14.76598*	4.62302	0.003	-24.1512	-5.3807
12 months	Oral B Gum and enamel (C)	ZACT Stain fighter (ZW)	-14.70278*	3.52178	0.000	-21.8524	-7.5532
		Colgate optic volcano mineral (CW)	7.04980	3.52178	0.053	-0.0998	14.1994
		Oral B 3D Fresh breathe (3DW)	-12.90626*	3.52178	0.001	-20.0558	-5.7567
		Thepthai herbal toothpaste(TW)	1.33121	3.52178	0.708	-5.8184	8.4808
	ZACT Stain fighter (ZW)	Oral B Gum and enamel (C)	14.70278*	3.52178	0.000	7.5532	21.8524
		Colgate optic volcano mineral (CW)	21.75257*	3.52178	0.000	14.6030	28.9022
		Oral B 3D Fresh breathe (3DW)	1.79652	3.52178	0.613	-5.3531	8.9461
		Thepthai herbal toothpaste(TW)	16.03399*	3.52178	0.000	8.8844	23.1836
	Colgate optic volcano mineral (CW)	Oral B Gum and enamel (C)	-7.04980	3.52178	0.053	-14.1994	0.0998
		ZACT Stain fighter (ZW)	-21.75257*	3.52178	0.000	-28.9022	-14.6030
		Oral B 3D Fresh breathe (3DW)	-19.95606*	3.52178	0.000	-27.1056	-12.8065
		Thepthai herbal toothpaste(TW)	-5.71858	3.52178	0.113	-12.8682	1.4310
	Oral B 3D Fresh breathe (3DW)	Oral B Gum and enamel (C)	12.90626*	3.52178	0.001	5.7567	20.0558
		ZACT Stain fighter (ZW)	-1.79652	3.52178	0.613	-8.9461	5.3531
		Colgate optic volcano mineral (CW)	19.95606*	3.52178	0.000	12.8065	27.1056
		Thepthai herbal toothpaste(TW)	14.23747*	3.52178	0.000	7.0879	21.3871
	Thepthai herbal toothpaste(TW)	Oral B Gum and enamel (C)	-1.33121	3.52178	0.708	-8.4808	5.8184
		ZACT Stain fighter (ZW)	-16.03399*	3.52178	0.000	-23.1836	-8.8844
		Colgate optic volcano mineral (CW)	5.71858	3.52178	0.113	-1.4310	12.8682
		Oral B 3D Fresh breathe (3DW)	-14.23747*	3.52178	0.000	-21.3871	-7.0879

*. The mean difference is significant at the 0.05 level.

Table S7. Dunn's post hoc test showed multiple comparison of ΔE_{00} among time points of each toothpaste.

Toothpaste	Time points	Test Statistic	Std. Error	Std. Test Statistic	Sig.
Oral-B gum and enamel (C)	2 weeks - 6 months	0.375	0.645	0.581	0.561
	2 weeks - 4 weeks	0.750	0.645	1.162	0.245
	2 weeks – 12 months	2.375	0.645	3.679	0.000
	6 months - 4 weeks	-0.375	0.645	-0.581	0.561
	6 months – 12 months	2.000	0.645	3.098	0.002
	4 weeks - 12months	1.625	0.645	2.517	0.012
ZACT Staing fighter (ZW)	2 weeks - 4 weeks	1.000	0.645	1.549	0.121
	2 weeks - 6 months	2.000	0.645	3.098	0.002
	2 weeks – 12 months	3.000	0.645	4.648	0.000
	4 weeks - 6 months	1.000	0.645	1.549	0.121
	4 weeks -12 months	2.000	0.645	3.098	0.002
	6 months -12 months	1.000	0.645	1.549	0.121
Colgate optic volcanic mineral (CW)	2 weeks - 4 weeks	0.250	0.645	0.387	0.699
	2 weeks - 6 months	1.000	0.645	1.549	0.121
	2 weeks -12 months	2.250	0.645	3.486	0.000
	4 weeks - 6 months	0.750	0.645	1.162	0.245
	4 weeks -12 months	2.000	0.645	3.098	0.002
	6 months -12 months	1.250	0.645	1.936	0.053
Oral B 3D Fresh breath (3DW)	2 weeks - 4 weeks	1.000	0.645	1.549	0.121
	2 weeks - 6 months	2.125	0.645	3.292	0.001
	2 weeks -12 months	2.875	0.645	4.454	0.000
	4 weeks - 6 months	1.125	0.645	1.743	0.081
	4 weeks -12 months	1.875	0.645	2.905	0.004
	6 months -12 months	0.750	0.645	1.162	0.245
Thepthai (TW)	2 weeks - 6 months	0.500	0.645	0.775	0.439
	2 weeks - 4 weeks	0.625	0.645	0.968	0.333
	2 weeks -12 months	2.375	0.645	3.679	0.000
	6 months - 4 weeks	-0.125	0.645	-0.194	0.846
	6 months -12 months	1.875	0.645	2.905	0.004
	4 weeks -12 months	1.750	0.645	2.711	0.007
